# Gintonin Binds to Reduced LPA4 Receptor Subtype in Human Cortical Neurons in Alzheimer’s Disease Brains

**DOI:** 10.3390/biom15020179

**Published:** 2025-01-26

**Authors:** Kyu-Sung Kim, Rami Lee, Inyeong Park, Sung-Hee Hwang, Yeshin Kim, Jae-Won Jang, Hyung-Seok Kim, Seong-Min Choi, Sang Jin Kim, Hwa Jin Cho, Ik-Hyun Cho, Jong-Hoon Kim, Do-Geun Kim, Seung-Yeol Nah

**Affiliations:** 1 Department of Brain Sciences, Daegu Gyeongbuk Institute of Science and Technology (DGIST), Daegu 42988, Republic of Korea; kks9286@kbri.re.kr; 2 Neuroimmunology Laboratory, Dementia Research Group, Korea Brain Research Institute, Daegu 41062, Republic of Korea; 3 Ginsentology Research Laboratory, Department of Physiology, College of Veterinary Medicine, Konkuk University, Seoul 05029, Republic of Korea; 4 Department of Pharmaceutical Engineering, College of Health Sciences, Sangji University, Wonju 26339, Republic of Korea; sunghhwang@sangji.ac.kr; 5 Department of Neurology, Kangwon National University Hospital, Chuncheon 24289, Republic of Korea; yeshins@gmail.com (Y.K.), light26@kangwon.ac.kr (J.-W.J.); 6 Department of Neurosurgery, Chonnam National University Medical School, Research Institute of Medical Sciences, Gwangju 61469, Republic of Korea; veritas@jnu.ac.kr; 7 Department of Neurology, Chonnam National University Medical School, Jebong-ro, Gwangju 61469, Republic of Korea; drchoism@lycos.co.kr; 8 Department of Neurology, Busan Paik Hospital, Inje University College of Medicine, Busan 47392, Republic of Korea; jsk120@hanmail.net; 9 Dementia and Neurodegenerative Disease Research Center, Inje University, Busan 47392, Republic of Korea; 10 Busan & Gyeongnam Reference Laboratory, Department of Pathology, Seegene Medical Foundation, Busan 48792, Republic of Korea; eunhasu101@hanmail.net; 11 Department of Convergence Medical Science, College of Korean Medicine, Kyung Hee University, Seoul 02447, Republic of Korea; ihcho@khu.ac.kr; 12 College of Veterinary Medicine, Biosafety Research Institute, Chonbuk National University, Iksan-City 54596, Republic of Korea; jhkim1@jbnu.ac.kr

**Keywords:** ginseng, gintonin, LPA4 receptor, human cortex, AD prevention

## Abstract

Ginseng, a traditional herbal medicine with a long history of use, is known to support human health, particularly by influencing brain function. Recent studies have identified gintonin, a lysophosphatidic acid (LPA) receptor ligand derived from ginseng, as a key bioactive. However, the specific LPA receptor subtypes targeted by gintonin in the human brain to exert its anti-Alzheimer’s (AD) effects remain unclear. This study aimed to elucidate the LPA receptor subtype targeted by gintonin in the human cortex. Using a fluorescent gintonin conjugate, we investigated receptor binding in cortical samples from healthy individuals (*n* = 4) and AD patients (*n* = 4). Our results demonstrated that fluorescent gintonin selectively binds to human cortical neurons rather than glial cells and that gintonin-binding sites are co-localized with the LPA4 receptor subtype. Furthermore, the expression of LPA4 receptors was significantly reduced in the cortical neurons of AD patients. These results suggest that the LPA4 receptor may serve as a novel histopathological marker for AD and represent a promising therapeutic target for gintonin-based prevention and treatment strategies.

## 1. Introduction

Alzheimer’s disease (AD) is the leading cause of dementia, and it poses a significant global health challenge, impacting both individuals and society, particularly in aging populations [1]. AD is characterized by the accumulation of amyloid plaques and tau neurofibrillary tangles [2,3], presenting with memory loss and progressive cognitive dysfunction [4]. Although several treatments have been developed to alleviate cognitive dysfunction, there are currently no drugs available that improve cognition or prevent the progression of AD [5]. Therefore, advancing our understanding of the molecular mechanisms underlying AD is of great clinical importance and could identify novel therapeutic targets.

The root of Panax ginseng C.A. Meyer has been traditionally used as a tonic in China, Japan, and Korea for over 2000 years and is now consumed worldwide [6]. Ginseng has long been believed to have tonic effects, such as energizing the body, enhancing brain function, elevating mood, and promoting longevity [7]. Currently, ginseng is recognized as both a functional food and herbal medicine.

Gintonin, a novel component of ginseng, was recently isolated [8,9]. It is a glycolipoprotein complex isolated using anion exchange chromatography to extract negatively charged components [8]. The main functionally active components of gintonin are lysophosphatidic acids (LPAs), which act as exogenous ligands for GTP-binding protein-coupled LPA receptors. The activation of these receptors by gintonin has been shown to have diverse biological effects, particularly in the nervous system [10]. The basic mechanism of action of gintonin involves inducing [Ca^2+^]_i_ transients in neuronal cells via high-affinity LPA receptor subtypes [8,9]. These [Ca^2+^]_i_ transients are coupled with increased acetylcholine synthesis and release and the induction of hippocampal neuronal precursor cell proliferation [11]. Additionally, gintonin stimulates the release of neurotransmitters (ATP and glutamate) and hormones (BDNF, dopamine, and norepinephrine) in vitro and ex vivo [12], and is associated with enhanced synaptic transmission [13]. The facilitation of synaptic transmission by gintonin has been linked to improved hippocampal learning and memory in mice [14].

In an AD animal model, the long-term oral administration of gintonin inhibited amyloid plaque accumulation in the cortex and hippocampus and alleviated cognitive dysfunction caused by amyloid plaques [15]. In in vitro studies, gintonin inhibits Aβ_1-42_ formation and release and protects neuronal cell death induced by Aβ_1-42_ [15]. In vitro and in vivo studies on gintonin-mediated blood–brain barrier (BBB) regulation have shown that the acute intravenous administration of gintonin opens the BBB and enhances donepezil delivery, a current AD medication, into the brain by 30% [16,17]. In addition, we demonstrated that gintonin, when conjugated with a fluorescent agent, enters the mouse brain and binds to brain cells, such as neurons and glial cells [17].

In clinical human studies, the long-term oral administration of a gintonin-enriched fraction (GEF) for 8 weeks improved cognitive function in patients with early AD in a pilot open-label trial without adverse effects [18]. Furthermore, Lee et al. showed that GEF-mediated improvement in cognitive function via the oral route was closely associated with the gintonin-mediated enhancement of BBB permeability in a dynamic contrast-enhanced MRI pilot study, consistent with findings in mice [19]. In a placebo-controlled double-blind study, GEF administration to subjects with subjective memory impairment (SMI) for 4 and 8 weeks (300 or 600 mg/day) significantly improved ADAS-Cog scores and performance on the Stroop Color and Word Test compared to that by placebo treatment [20]. These findings demonstrate that gintonin exhibits preventive effects against AD through LPA receptor signaling in in vitro, in vivo AD animal models, and clinical studies. However, the specific human LPA receptor subtype that gintonin binds to in patients with early AD and SMI to exert its cognition-enhancing effects is not known.

In this study, we first examined the cell types in the cortices of healthy subjects and patients with AD that gintonin binds to. Next, we investigated the LPA receptor subtypes that gintonin binds to in the human cortices of both healthy subjects and patients with AD. We further discuss the potential of the human LPA4 receptor subtype as a novel histopathological marker for AD and suggest that gintonin could be a promising candidate for AD prevention and treatment by targeting this receptor subtype.

## 2. Materials and Methods

### 2.1. Materials

Gintonin was prepared from Panax ginseng C.A. Meyer ginseng using a previously reported method involving anion-exchange affinity chromatography [21]. For the in vitro studies, gintonin was dissolved in dimethyl sulfoxide (DMSO). Flamma 675 vinylsulfone (FPR675) was purchased from BioActs (Incheon, Republic of Korea). The FPR675-gintonin conjugate (fluorescent gintonin) was constructed following the BioActs protocol [22].

### 2.2. Human Brain Tissues from Healthy Participants and Patients with AD

This study included human subjects and was approved by the Konkuk University Institutional Review Board (IRB) (7001355-202103-E-135). All human tissues were obtained from IRB-approved brain banks. Tissue blocks were requested to maintain structural integrity. Nine frozen tissue samples (0.2 cm) were donated by the Korean Brain Bank Network (KBBN) and included both non-diseased (ND) and Alzheimer’s disease (AD) brains. Participants consisted of three males and two females in the ND group and four males in the AD group, matched for age, sex, and PMI.

### 2.3. Immunofluorescence Assay

Frozen human brain tissue sections were mounted on glass slides. The slides were fixed in 4% paraformaldehyde for 20 min at room temperature. Permeabilization was achieved with 1% Triton X-100 in PBS for 30 min. After rinsing with PBS, the slides were blocked with 5% normal goat serum (NGS) in PBS for 1 h at room temperature to minimize nonspecific binding. The primary antibodies used were mouse anti-NeuN (1:500, #MAB377, EMD Millipore, Billerica, MA, USA), rabbit anti-Iba1 (1:500, 019-19741, Wako, Osaka, Japan), FITC-anti-CD11b (1:100, 101206, Bio Legend, San Diego, CA, USA), mouse anti-mouse GFAP (1:500, 3670S, CST, Danvers, MA, USA), rabbit anti-LPAR1 (1:500, ALR-031, Alomone labs, Jerusalem, Israel), rabbit anti-LPAR2 (1:500, ALR-032, Alomone labs), rabbit anti-LPAR3 (1:500, ab23692, Abcam, Cambrighe, UK), rabbit anti-LPAR4 (1:500, ALR-034, Alomone labs), rabbit anti-LPAR5 (1:500, ALR-035, Alomone labs), and rabbit anti-LPAR6 (1:500, ALR-036, Alomone labs). The secondary antibodies were conjugated to Alexa Fluor 488, 568, and 647 (1:500; Invitrogen, Waltham, MA, USA). For gintonin (GT) detection, GT was conjugated to FPR^TM^ 675 (Bioacts, Incheon, Republic of Korea). DAPI (17984-24, Electron Microscopy Science, Hatfield, PA, USA) was applied to the tissue, and a cover slip was mounted.

### 2.4. Co-Localization Analysis

A co-localization analysis was performed using the ImageJ Version 1.54m software (NIH, Bethesda, MD, USA). The images were first converted to 8-bit grayscale, and regions of interest (ROIs) were selected based on the relevant signal thresholds. For quantification, the “Coloc 2” plugin was utilized to calculate the Pearson correlation coefficient (PCC) and Manders’ overlap coefficients (M1 and M2), which measure the degree of co-localization between the selected channels. Prior to analysis, thresholds were set automatically using the Costes method to minimize background noise. The results were visualized and statistically analyzed to determine the extent of co-localization between the respective markers.

### 2.5. Western Blotting

The brain tissues were homogenized and lysed using RIPA buffer containing 1% phosphatase and protease inhibitor cocktail. Equal amounts of protein were loaded onto an SDS-PAGE gel and separated using a Bio-Rad electrophoresis system. The proteins were transferred to nitrocellulose (NC) membranes. The blots were blocked with 1% BSA in TBST buffer for 15 min at 4 °C and incubated with primary antibodies diluted in 1% BSA in TBST buffer overnight at 4 °C. The primary antibodies used were LPAR1 (1:2000, ALR-031, Alomone Labs), LPAR2 (1:2000, ALR-032, Alomone Labs), LPAR3 (1:2000, ab23692, Abcam), LPAR4 (1:2000, ALR-034, Alomone Labs), LPAR5 (1:2000, ALR-035, Alomone Labs), LPAR6 (1:2000, ALR-036, Alomone labs), and β-actin (1:2000, 3700s, CST). After washing with TBST, the blots were incubated with horseradish peroxidase-conjugated anti-rabbit and anti-mouse IgGs (Jackson ImmunoResearch Laboratory, West Grove, PA, USA) in 5% skim milk/TBST for 1 h at room temperature. The blots were developed using Lumigen ECL ultra-solution (Lumigen, Southfield, MI, USA), and signals were detected using the ChemiDoc^TM^ MP Imaging System and analyzed using the ImageJ software.

### 2.6. Statistical Analysis

All the experiments were repeated at least three times. Differences between the two groups were analyzed using Student’s *t*-test. Statistical analyses were performed with the GraphPad Prism 10 software (GraphPad Software, San Diego, CA, USA).

## 3. Results

### 3.1. Gintonin-Binding Sites Are Co-Localized with NeuN, a Neuronal Marker Protein

**Figure 1:**
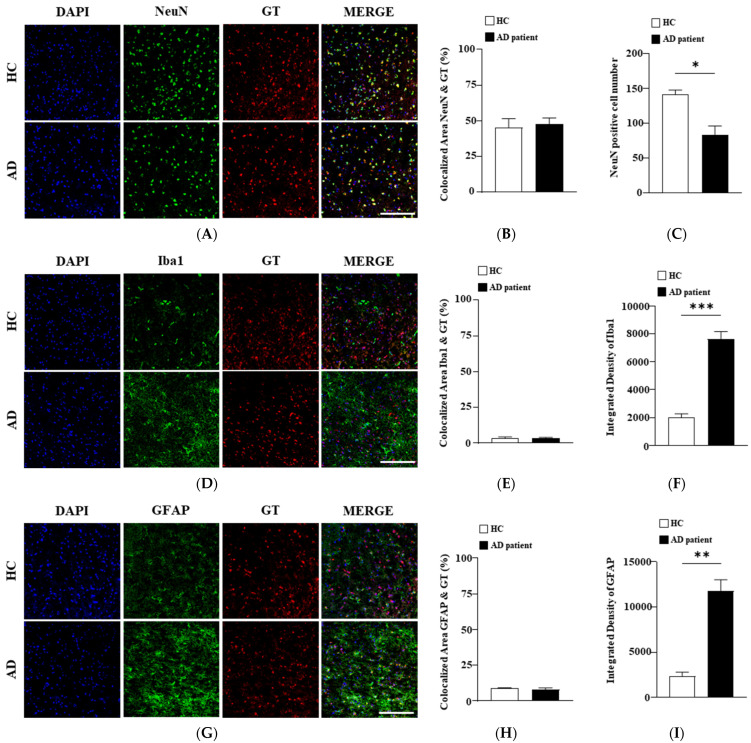
Identification of gintonin (GT)-binding cells in the cortices of the healthy controls (HCs) and Alzheimer’s disease (AD) patients. (**A**) Representative confocal images showing DAPI (blue), NeuN (a neuronal marker, green), and GT (red) in the brains of the HCs and patients with AD. Scale bar = 200 μm. (**B**) Quantitative analysis of the co-localization between NeuN and GT and (**C**) NeuN positive cell number. (**D**) Representative confocal images showing DAPI (blue), Iba1 (a microglia marker, green), and GT (red) in the brains of the HCs and patients with AD. Scale bar = 200 μm. (**E**) Quantitative analysis of the co-localization between Iba-1 and GT and (**F**) integrated density of Iba1. (**G**) Representative confocal images showing GFAP (an astrocyte marker, green) and GT (red) in the brains of the HCs and patients with AD. (**H**) Quantitative analysis of the co-localization between GFAP and GT and (**I**) integrated density of GFAP. Scale bar = 200 μm. Statistical significance was determined using a *t*-test. Data are presented as mean ± SEM. * *p* < 0.05, ** *p* < 0.01 and *** *p* < 0.001.

In our previous study, we demonstrated that the systemic administration of gintonin enabled it to cross the BBB and bind to neuronal cells. However, it remained unclear which specific cell types in the human cortex gintonin binds to [17,22]. In this study, we used three different antibodies: NeuN, a marker for neurons; GFAP, a marker for astrocytes; and Iba-1, a marker for microglia. We first confirmed the gintonin-binding cells by identifying their respective marker proteins (NeuN, GFAP, or Iba-1). As shown in Figure 1A,B, fluorescent gintonin binds to human cortical tissue, with binding sites co-localized with NeuN antibody-binding sites in both healthy control (HC) and patients with AD cortices at over 40% (Figure 1A,B). However, the number of NeuN-expressing neurons was significantly reduced in patients with AD compared to healthy cortices (Figure 1C). Notably, gintonin-binding sites showed minimal co-localization with GFAP and Iba-1 marker proteins (less than 10%; Figure 1D,E, Figure 1G,H), although the numbers of Iba1- and GFAP-positive cells were significantly increased in patients with AD compared to healthy cortices (Figure 1F,I). These results indicate that gintonin preferentially binds to human cortical neurons rather than astrocytes or microglia (Figure 1). Additionally, astrocytes and microglia in patients with AD, as indicated by GFAP and Iba-1 staining, were more activated compared to HC. Thus, GFAP and Iba1 protein expression levels were higher in patients with AD than in HC (Figure 1F,I).

### 3.2. Gintonin Binds to Neurons Expressing the LPA4 Receptor Subtype

**Figure 2:**
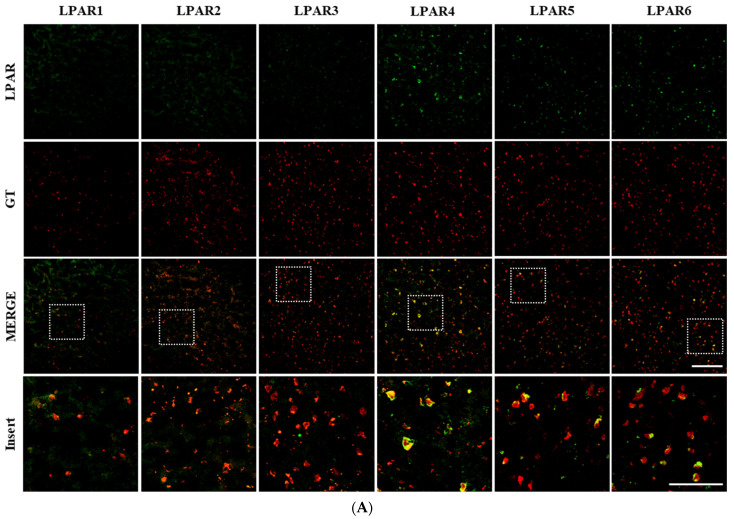

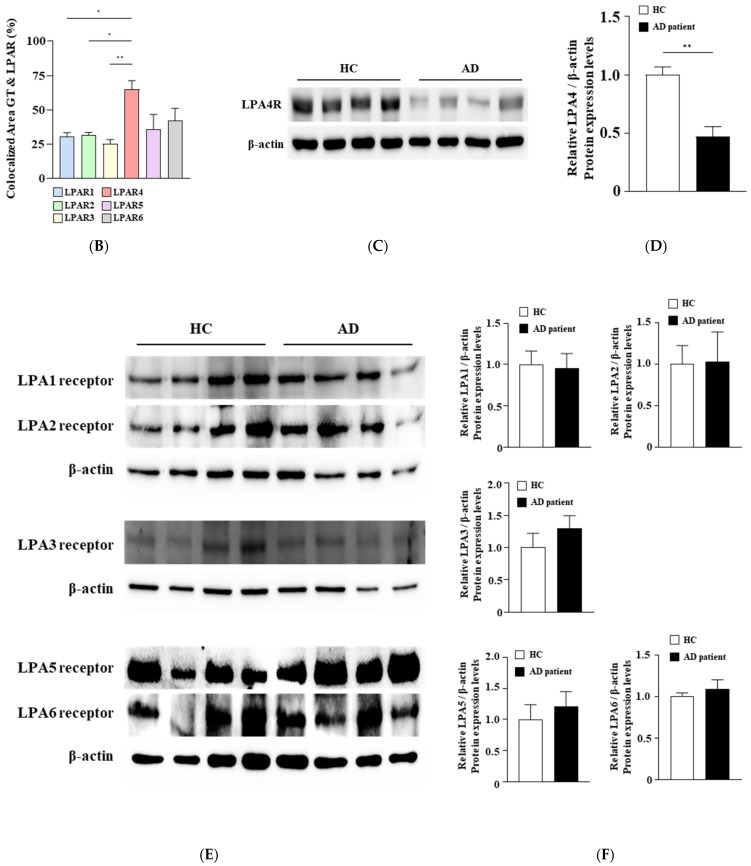
Co-localization of the gintonin (GT)-binding site with the LPA4 receptor subtype and a selective reduction in LPA4 receptor subtype expression levels in patients with AD. (**A**) Representative confocal images of the LPA receptor subtypes (LPAR1, LPAR2, LPAR3, LPAR4, LPAR5, and LPAR6; green) and GT (red) in the cortices of the HCs and patients with AD. Scale bar = 200 μm. The *insert* images focus on the co-localization between gintonin (GT) and the LAP receptor subtypes. Scale bar = 100 μm. (**B**) Quantitative analysis of the co-localization between the GT and LPA receptor subtypes. (**C**–**F**) Western blot analysis of the LPA receptor subtypes in brain tissue from the HCs and patients with AD. The results indicate a significant decrease in LPAR4 expression in patients with AD, whereas no significant differences were observed for the other LPA receptor subtypes. Statistical significance was determined using a *t*-test. Data are presented as mean ± SEM. * *p* < 0.05, ** *p* < 0.01. Original Western blot images can be found in Figure S1.

There are currently six known LPA subtypes [23]. Frugier et al. (2011) demonstrated low expression of LPA1-3 receptors in the normal human brain through immunohistochemistry, while LPA4-6 receptor subtypes were not reported [24]. Our current study aligns with this previous study, showing low expression of LPA1-3 receptors in the normal human brain. However, LPA4-6 receptor subtypes, particularly LPA4, were expressed at higher levels in the human cortex (Figure 2A). Given that the biological effects of gintonin are mediated through LPA receptor signaling, we examined which subtypes gintonin binds to in the human brain cortices of both the HCs and patients with AD. As shown in Figure 2A, the gintonin-binding sites co-localized with the LPA4 receptor antibody-binding sites in both the HC and AD cortices (Figure 2A,B). The binding sites of fluorescent gintonin were partially or fully overlapping with the LPA4 receptor antibody sites (Figure 2A, *insert*). Additionally, the gintonin-binding sites were partially co-localized with LPA5 and LPA6 receptors, but not LPA1-3, in both HCs and patients with AD. The percentage of co-localization was highest with LPA4 at 65%, followed by LPA6 at 42% and LPA5 at 36%, indicating that the LPA4 receptor subtype is the primary binding site for gintonin in human cortical neurons.

We also compared the cortical protein expression levels of LPA receptor subtypes between the HCs and patients with AD using Western blotting. As shown in Figure 2C–F, there was a significant reduction in LPA4 receptor protein expression in the AD patients compared to HC. No significant differences were observed for the other five LPA receptor subtypes between the two groups. These findings suggest that AD progression may selectively affect LPA4 receptor expression among the six LPA receptor subtypes.

### 3.3. Comparison of LPA4 Receptor Subtype Expression in Neurons, Astrocytes, and Microglia Between HCs and Patients with AD

**Figure 3:**
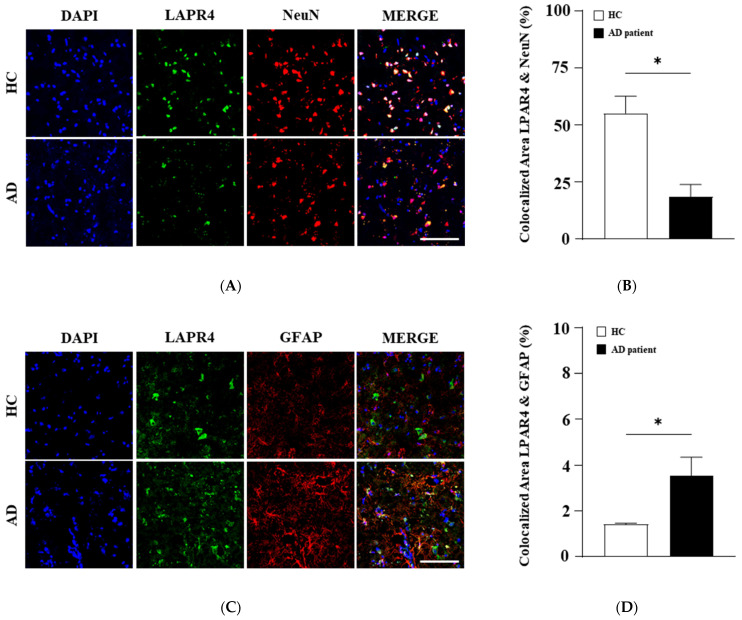

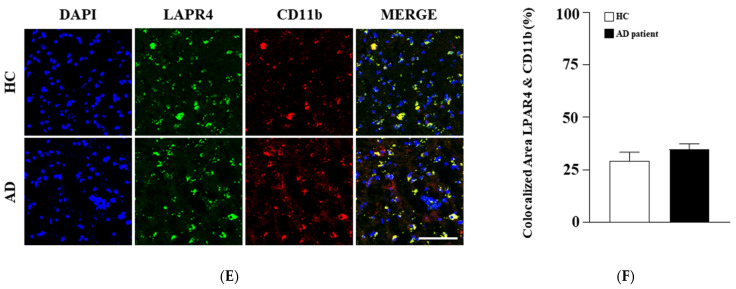
The relationship between LPA4 receptor subtype expression in the cortices of HCs and patients with AD. (**A**) Representative confocal images showing DAPI (blue), LPAR4 (green), and NeuN (red) in the cortices of HCs and patients with AD. Scale bar = 100 μm. (**B**) Quantitative analysis of the co-localization between the LPA4 receptor subtype and NeuN. (**C**) Representative confocal images showing DAPI (blue), LPAR4 (green), and GFAP (red) in the brains of HCs and patients with AD. Scale bar = 100 μm. (**D**) Quantitative analysis of the co-localization between the LPA4 receptor subtype and GFAP. (**E**) Representative confocal images showing DAPI (blue), LPAR4 (green), and Iba1 (red) in the cortices of HCs and patients with AD. Scale bar = 100 μm. (**F**) Quantitative analysis of the co-localization between the LPA receptor subtype and Iba 1. Statistical significance was determined using a *t*-test. Data are presented as mean ± SEM. * *p* < 0.05.

To investigate changes in LPA4 expression in different cell types, we examined co-localization with NeuN (neurons), GFAP (astrocytes), and Iba-1 (microglia) antibodies. As shown in Figure 3A, LPA4 receptor expression was lower in the cortical neurons of patients with AD compared to HCs, as determined by co-localization with NeuN (Figure 3A,B). Interestingly, LPA4 co-localization in cortical astrocytes increased in patients with AD and correlated with elevated GFAP protein expression levels (Figure 3C,D). However, there were no significant changes in LPA4 expression in microglial cells between AD and HC (Figure 3E,F). These results suggest that the reduction in neuron number in patients with AD may be associated with decreased LPA4 expression levels.

## 4. Discussion

AD is prevalent worldwide and is predicted to rapidly increase with increasing longevity in an aging society [25]. However, the current medications do not address the underlying pathophysiology of AD and provide only modest, temporary symptomatic relief while having significant side effects [26]. The characteristic brain markers of AD dementia include the accumulation of amyloid plaques and neurofibrillary tangles, which are intracellular aggregates of hyperphosphorylated tau proteins with dead neurons [27]. In addition, the impairment of the brain’s cholinergic system leads to reduced acetylcholine levels and an increase in acetylcholinesterase in patients with AD [28]. Other histopathological markers of AD, however, remain largely unknown. In the present study, we could also observe reductions in neurons and increases in astrocytes and microglia, which are typically well-known brain histopathological symptoms (Figure 2).

On the other hand, LPA, a pleiotropic lipid mediator, plays a crucial role in cell proliferation, survival, and migration and functions as a growth factor-like molecule in the development and maintenance of the nervous system via regulation of G protein-coupled LPA receptors [29]. The LPA1 receptor subtype, for instance, is important for early cortical development in animal models [30]. Although increased levels of LPA2 receptor mRNA have been observed in the adult cortex following traumatic brain injury [24], the roles of the six LPA receptor subtypes in the context of AD in humans are still poorly understood. In this study, we present evidence suggesting that the GTP-binding-coupled LPA4 receptor subtype is involved in novel AD-related pathophysiology. Specifically, LPA4 receptor expression was found to be selectively reduced in the cortices of patients with AD compared to HC.

The LPA receptor subtypes can be classified into two categories [31]: the Edg family (LPA1 to LPA3) and the non-Edg family (LPA4 to LPA6) [31]. Previous research demonstrated that fluorescent gintonin binds to mouse brain cells [16,32]. Further work showed that biotinylated gintonin primarily binds to LPA1 and LPA6 receptor subtypes in human prostate cancer cell (PC3) membranes, inducing [Ca^2+^]_i_ transients without interference from biotin conjugates [33]. In this study, we observed that fluorescent gintonin predominantly binds to non-Edg LPA receptor subtypes, in the order of LPA4 >> LPA6 > LPA5, in human cortical neurons (Figure 2A,B). Although the LPA1 and LPA2 subtypes were identified earlier in mouse brain development than the LPA4 to LPA6 subtypes, our findings suggest that non-Edg family LPA receptors, such as LPA4, may play a more significant role in AD pathophysiology than the Edg family subtypes (Figure 2 and Figure 3). This indicates that LPA4 receptor subtypes are likely more involved in AD than the Edg family subtypes in humans.

Reports on the roles of the LPA4 receptor subtype in nervous and non-nervous systems remain limited, particularly with respect to signaling through Gαs and Gαi proteins. Gαs activates adenylyl cyclase, converting ATP to cAMP, whereas the Gαi protein inhibits this pathway [34]. For example, LPA4 receptor activation can suppress LPA1 receptor-mediated cell motility [35]. During development, LPA4 receptor activation has been shown to increase blood and lymphatic vessel formation, support lymphocyte transmigration, and enhance hematopoiesis via bone marrow stromal cells [36,37]. Additionally, experimental atherosclerosis is reduced in mice lacking the LPA4 receptor subtype [38]. In the nervous system, only one study has reported that LPA4 receptor expression changes during prenatal and postnatal stages, with different distributions in the hippocampus and cortex of mice [34]. In humans, the role of LPA4, particularly in the AD brain, remains unclear. Future research should focus on exploring the correlation between LPA4 receptor subtype impairment and cognitive dysfunction in patients with AD, in addition to cholinergic system impairment.

Accumulating evidence supports the anti-AD potential of gintonin, a novel LPA receptor ligand derived from ginseng, in both in vitro and in vivo AD models [11,15,39]. However, prior to this study, it was unclear which specific types of human brain cells gintonin binds to and which LPA receptor subtypes are involved. This study addresses these questions by demonstrating that gintonin binds primarily to adult human cortical neurons, with binding sites co-localized with the LPA4 receptor subtype, and not the other five LPA receptor subtypes (Figure 1 and Figure 2). Furthermore, we showed that among the six LPA receptor subtypes, only the LPA4 receptor subtype expression level among six LPA receptor subtypes was selectively and significantly reduced in patients with AD compared to that in HC (Figure 2). This selective reduction suggests that the LPA4 receptor subtypes may play important pathophysiological roles in AD incidence and progression with relevant reductions compared to the other LPA4 receptor subtypes (Figure 2 and Figure 3), although the exact mechanisms remain to be determined. Our findings suggest that the LPA4 receptor subtype may serve as a novel biomarker for AD pathology. Further investigation is needed to establish whether gintonin intake can prevent AD onset or improve cognitive dysfunction in early patients with AD through modulation of the brain’s LPA4 receptor signaling pathway.

In conclusion, our study shows that gintonin, a bioactive LPA receptor ligand from ginseng, binds to human brain cortical neurons, with binding sites co-localizing with the LPA4 receptor subtype, which is reduced in patients with AD. Given the previous findings of gintonin-mediated cognitive improvements in patients with early AD and SMI, we hypothesize that gintonin’s potential to prevent AD may be mediated through the regulation of the LPA4 receptor signaling pathway. In addition, our study provides evidence that the LPA4 receptor subtype can be a novel histopathological marker in AD and a promising target for drug development, including therapies using gintonin or other LPA4 receptor subtype agonists.

## Data Availability

The data are contained within the article.

## Supplementary Materials

The following supporting information can be downloaded at: https://www.mdpi.com/article/10.3390/biom15020179/s1, Figure S1: Original Western Blot images of Figure 2C,E.
