# Supplementary material for: Gintonin Binds to Reduced LPA4 Receptor Subtype in Human Cortical Neurons in Alzheimer’s Disease Brains
Source: Biomolecules. 2025 Jan 26;15(2):179. doi: 10.3390/biom15020179 (PMC11853258; doi:10.3390/biom15020179)
Supplement: Supplementary file 1 [file biomolecules-15-00179-s001.zip › biomolecules-3314944-supplementary.pdf]

Supplementary Materials

Uncropped western blotting raw data below:

Figure 2. C LAPR4 & β-actin Western blot

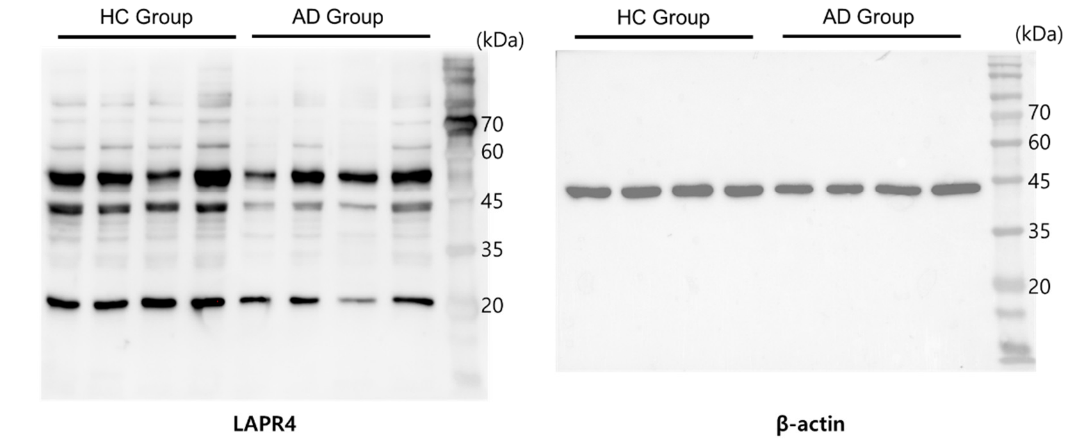

Figure 2. E LAPR1 & β-actin Western blot

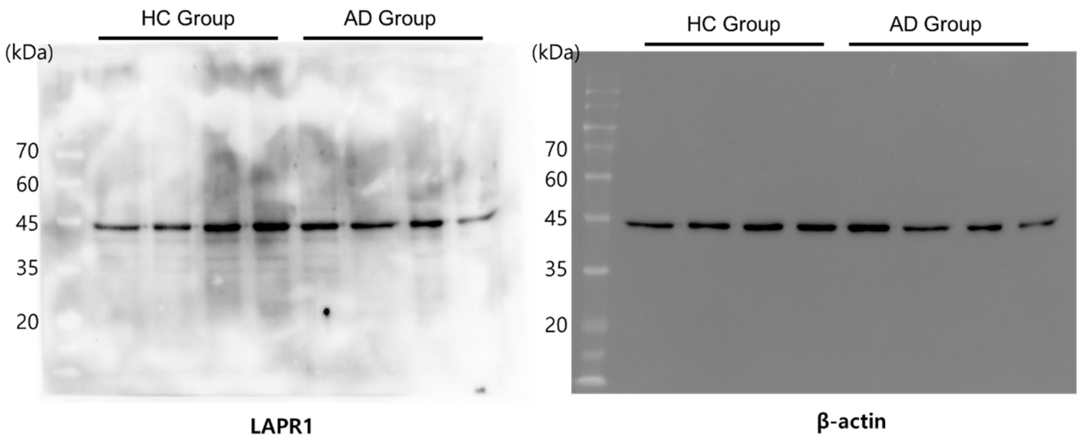

Figure 2. E LAPR2 & β-actin Western blot

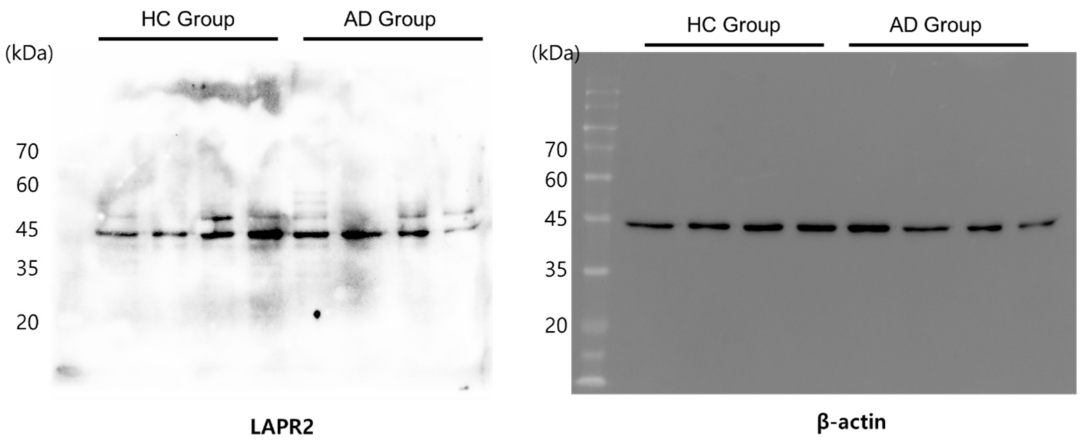

Figure 2. E LAPR3 & β-actin Western blot

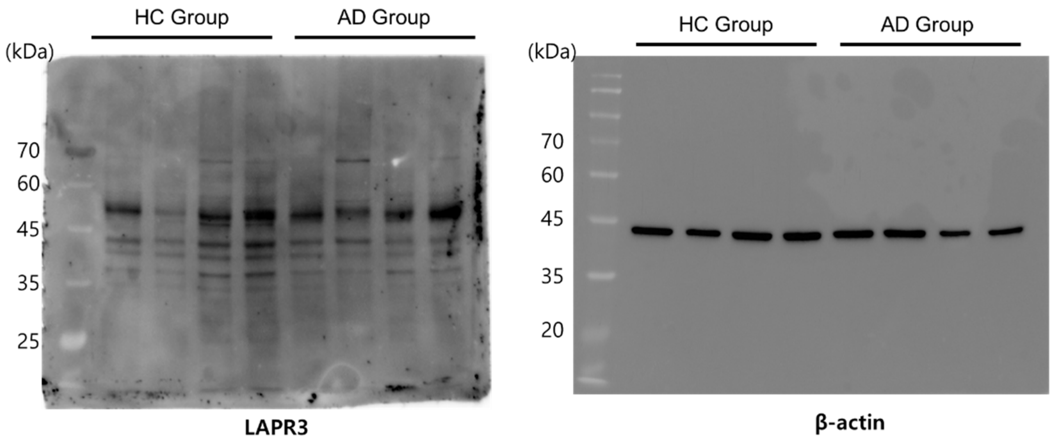

Figure 2. E LAPR5 & β-actin Western blot

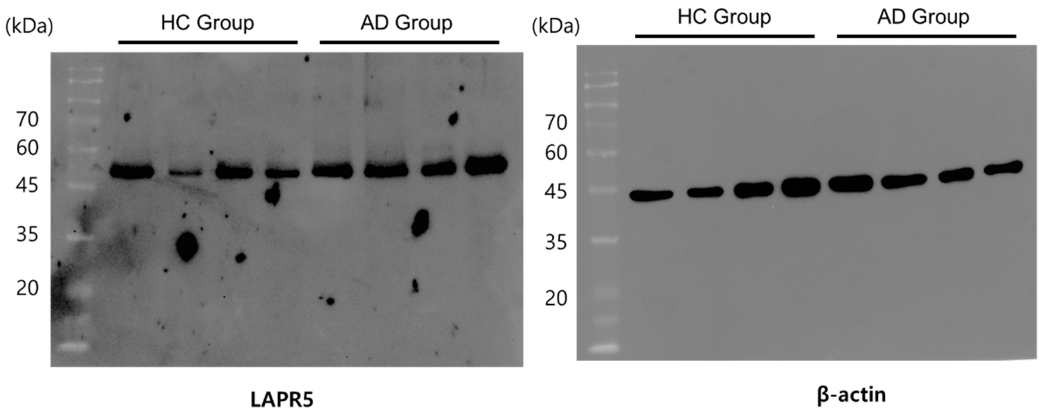

Figure 2. E LAPR6 & β-actin Western blot

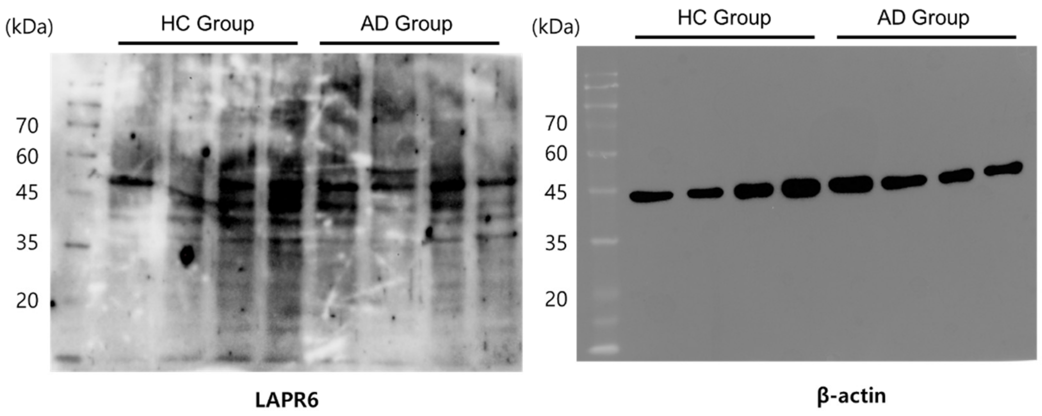

Figure S1. Original Western Blot images of Figure 2C,E.
